# Supplementary material for: Patterns of Intron Gain and Loss in Fungi
Source: PLoS Biol. 2004 Nov 30;2(12):e422. doi: 10.1371/journal.pbio.0020422 (PMC532390; doi:10.1371/journal.pbio.0020422)
Supplement: Table S1 — Also available at http://genes.mit.edu/NielsenEtAl/. (4.3 MB ZIP). [file pbio.0020422.st001.zip › NielsenEtAl/html/299.html]

AN7430.1.NCU07156.1.MG00253.1.FG09984.1


```
 CLUSTAL W (1.82) Multiple Sequence Alignments - Introns Inserted


Sequence 1: NCU07156.1	553 aa
Sequence 2: FG09984.1	549 aa
Sequence 3: MG00253.1	555 aa
Sequence 4: AN7430.1	553 aa
Alignment Length: 565 aa
Number Identitical Residues: 351 aa
Alignment Score (without introns) 15687


MG00253.1 	MPKVYLLDYVAGNVRSLVNAIEKVGFEVEWVKTPEEVAKAD0KLILPGVGHFGHCLSQLS
NCU07156.1	MPTVHLLDYVAGNVRSLVNAIERVGYSVEWVRSPEEVANAD0KLILPGVGHFGHCLSQLA
FG09984.1 	MPTVHLLDYVAGNIRSLVNAIEKCGYEVEWVRSPEEVPNAE0KLILPGVGHFGHCLSQLS
AN7430.1  	MPTVHLLDYVAGNVRSLVNAINKVGYEVEWVRSPSDLKDVE0KLILPGVGHFGHCLSQLS
          	**.*:********:*******:: *:.****::*.:: ..: *****************:

MG00253.1 	KAGFLPAIQKHIADGKSFMGICVGMQAIFSGSSEDPACPGLGLIKGSLDRFNDSTKAVPH
NCU07156.1	SAGYMAPIRAHIESGKPFMGICVGLQALFEGSSEDPNCPGLGVLPGKLDRFDDTSKSVPH
FG09984.1 	QAGYLEPIKKHIADGKPFFGVCVGLQALFQGSVEDPDIPGLGVVPATLDRFDDSSKSVPH
AN7430.1  	SGGYLQPIREHIASGKPFMGICVGLQSLFESSEEDPNIPGLGTIPARLRKFDAKTKSVPH
          	..*:: .*: ** .**.*:*:***:*::*..* ***  **** : . * :*: .:*:***

MG00253.1 	IGWNDATITTTTQPNSNNEPSTLLDLRPSSKYYYVHSYKYPYVPGELEAQGWTVATGVYG
NCU07156.1	IGWNDASCPTN---------PNLFGLNPDSKYYYVHSYKMPYTKGQLESQGWAVATGVYG
FG09984.1 	IGWNNAFTAG----------KAMYDLQPDSKYYYVHSYKCPYKPGELESQGWAVATGTYG
AN7430.1  	IGWNSATDTRIDSTGG----QTFYGLSPSSKYYYVHSYAAPYEPGILEKDGWLVATASYG
          	****.*  .   ....      : .* *.*********  **  * ** :** ***. **

MG00253.1 	EETFVGAVARGNVLVTQFHPEKSGVAGLRVLRSFLDPSGAASLG---SAAVAPR----NP
NCU07156.1	GETFIGAIAKGNVMATQFHPEKSGVAGLRVIRAFLDGSGAAALASHPPQEVPNAGDDALV
FG09984.1 	AETFVGAIAKGNIYATQFHPEKSGAAGLRTLRAFLTGEGLKTLGNVVDEAVANA---PIK
AN7430.1  	EEKFIGAIARDNIFATQFHPEKSGQAGLRTLRAFLDGAQLHSVT--LEDSILTG------
          	 *.*:**:*:.*: .********* ****.:*:**      ::       :         

MG00253.1 	VPAGLTRRVIACLDVRTNDAGDLVVTKGDQYDVREK-SSDRG--VRNLGKPVDLAKKYYE
NCU07156.1	SKEGLTRRVIACLDVRTNDQGDLVVTKGDQYDVREK-SDDRN--VRNLGKPVEMARKYYE
FG09984.1 	FEDSLTRRVIACLDVRTNDQGDLVVTKGDQYDVREK-GDDRN--VRNLGKPVEMAKRYYE
AN7430.1  	EKNGLTRRIIACLDVRTNDVGDLVVTKGDQYDVREKDGADAGGQVRNLGKPVDMAKKYYE
          	   .****:********** ****************.. * ...********::*::***

MG00253.1 	QGADEVTFLNITSFRDCPLADLPMLEVLRLTSETVFVPLTVGGGIRDTVDVDGTKVSALD
NCU07156.1	QGADEVTFLNITSFRDCPVADLPMLEILRLTSKTVFVPLTVGGGIRDTVDTDGTKVSALE
FG09984.1 	SGADEVTFLNITSFRDCPVADLPMLEIVRQTSRTVFVPLTIGGGIRDTVDTDGTKVSALE
AN7430.1  	QGADEVTFLNITSFRNCPLADLPMLEILRRTSETVFVPLTIGGGIRDTVDTDGTHIPALD
          	.**************:**:*******::* **.*******:*********.***::.**:

MG00253.1 	VASMYFRSGADKVSIGSDAVTAAEEYYAAGKTLSGSTAIEQISRAYGNQAVVVSVDPKRV
NCU07156.1	IATMYFQSGADKVSIGSDAVIAAEEYYASGKTLFGNTAIEQISKAYGNQAVVVSVDPKRV
FG09984.1 	IATMYFKSGADKVSIGSDAVLAAEEYYSLGRKLFGNTAIEQISRAYGNQAVVVSVDPKRV
AN7430.1  	VASMYFKSGADKVSIGSDAVVAAEDYYAAGKVLSGKTAIETISKAYGNQAVVVSVDPKRV
          	:*:***:************* ***:**: *: * *.**** **:****************

MG00253.1 	YVASPEATRHATIQTAFPGPNGEAYCWYACTAKGGRETRDVDVVELTQAVEAMGAGEILL
NCU07156.1	YVPKPDATRHHTVKTSYPGPKGEEYCWYACTIKGGRETRDLDVVELTQAVEAMGCGEILL
FG09984.1 	YVPKPDATRHNIVKTQFPGPKGEEYCWYACTIKGGRETRDMDVVELTQAVEAMGTGEILL
AN7430.1  	YVSQPEDTKHRTIETKFPNAAGQNFCWYQCTIKGGRETRDLDVCQLVQAVEAMGAGEILL
          	**..*: *:*  ::* :*.. *: :*** ** ********:** :*.******* *****

MG00253.1 	NCIDKDGTNSGFDLELIAQVKAAVKIPVIASSGAGNPGHFEEVFEKTTTDAALGAGI~FH
NCU07156.1	NCIDKDGTNSGFDLELIRQVKAAVRIPVIASSGAGNPGHFEEVFRETTTDAALGAGM~FH
FG09984.1 	NCIDKDGTNSGFDFELINQVKGAVKIPVIASSGAGNPSHFEDVFQKTPTDAALGAGM~FH
AN7430.1  	NCIDKDGSNSGFDLELINHVKAAVKIPVIASSGAGMPKHFEEVFDQTTTDAALGAGM0FH
          	*******:*****:*** :**.**:********** * ***:** :*.********: **

MG00253.1 	RGEYTVTQVKDHLATKGLLVRQFEGEL
NCU07156.1	RGEYTVQQVKEELKARGLVVRQFEGDL
FG09984.1 	RGEYTVKQVKDYLQEKGLKVRQFEEGF
AN7430.1  	RGEYTVGEVKQYLEDRGFLVRRFEPDV
          	****** :**: *  :*: **:**  .
```
